# Macrophages and Extracellular Matrix in Breast Cancer: Partners in Crime or Protective Allies?

**DOI:** 10.3389/fonc.2021.620773

**Published:** 2021-02-24

**Authors:** Claire Deligne, Kim S. Midwood

**Affiliations:** Kennedy Institute of Rheumatology, Nuffield Department of Orthopaedics, Rheumatology and Musculoskeletal Sciences, University of Oxford, Oxford, United Kingdom

**Keywords:** extracellular matrix, macrophages, breast cancer, tumor microenvironment, immunotherapy, immune infiltrate

## Abstract

Solid cancers such as breast tumors comprise a collection of tumor, stromal and immune cells, embedded within a network of tumor-specific extracellular matrix. This matrix is associated with tumor aggression, treatment failure, chemo- and radio-resistance, poor survival and metastasis. Recent data report an immunomodulatory role for the matrix in cancer, *via* the creation of niches that control the migration, localization, phenotype and function of tumor-infiltrating immune cells, ultimately contributing to escape of immune surveillance. Macrophages are crucial components of the immune infiltrate in tumors; they are associated with a poor prognosis in breast cancer and contribute to shaping the anti-tumor immune response. We and others have described how matrix molecules commonly upregulated within the tumor stroma, such as tenascin-C, fibronectin and collagen, exert a complex influence over macrophage behavior, for example restricting or enhancing their infiltration into the tumor, and driving their polarization towards or away from a pro-tumoral phenotype, and how in turn macrophages can modify matrix production in the tumor to favor tumor growth and metastasis. Targeting specific domains of matrix molecules to reinstate an efficient anti-tumor immune response, and effectively control tumor growth and spread, is emerging as a promising field offering a new angle for cancer therapy. Here, we review current knowledge on the interactions between tumor-associated macrophages and matrix molecules that occur within the tumor microenvironment of breast cancer, and discuss how these pathways can be targeted for new immunotherapies for hard to treat, desmoplastic tumors.

## Introduction

The extracellular matrix (ECM) is a complex network of secreted molecules that, in healthy conditions, serves to define tissue architecture and stiffness, and program cell behavior including supporting cell adhesion, survival and migration. The matrix comprises collagens, proteoglycans and glycoproteins (including fibronectin, laminin, osteopontin, tenascin-C), along with a variety of matrix-associated molecules such as glycosaminoglycans, enzymes such as proteases, cross-linkers and kinases, and soluble factors such as chemokines, growth factors and cytokines (1). The study of the stroma during neoplasia reveals a deeply reorganized composition compared to healthy tissues, at both the cellular and molecular levels. In particular, tumors comprise a highly heterogeneous and dysregulated ECM network, embedding tumor cells and cancer-associated fibroblasts (CAF), as well as newly developed blood vessels (2). In breast cancer, ECM accumulation and desmoplasia in general are associated with a poor prognosis (3), and increased matrix deposition can predict breast tumor formation (4). Well-studied changes in the breast TME include abnormal matrix molecule expression, permanent remodeling, destruction by proteolytic enzymes and concomitant repair (5). Moreover, changes in individual matrix constituents have also been associated with breast cancer aggressiveness and metastasis. For example, tenascin-C, a protein which is not detected in most healthy adult tissues is notably re-expressed during tumorigenesis (6), deposition of collagen types I, III and V is particularly affected, with progressive fibril linearization and thickening over time during breast carcinogenesis (7–9), and epithelial upregulation of the ubiquitous matrix glycoprotein fibronectin (10) as well as enrichment of specific splice variants containing the oncofetal extra domain A (EDA) or extra domain B (EDB) (11) are observed. At the functional level, the unique matrix composition of tumors can influence all aspects of carcinogenesis, from angiogenesis, EMT, metastasis to immune surveillance (12). However, the molecular mechanisms defining cell-matrix interactions in the TME are not yet completely understood, highlighting the need for a better understanding of the role of the ECM during cancer evolution.

Macrophages have a central role in cancer immune surveillance in general (13) and in breast cancers in particular, where they are associated with a poor prognosis (14) and with negative hormone receptor status and malignant phenotype (15). However the prognostic role of tumor-associated macrophages (TAM) is not as clear cut as reported for other immune subsets, such as Th1 or Treg cells (16), as TAM may be also associated with a positive outcome in some cancers like colorectal carcinoma (14). This discrepancy may be explained by the extraordinary phenotypic plasticity of these cells, which are easily modified depending on local stimuli from the tumor microenvironment (TME). TAMs with a “M1-like” phenotype are characterized by tumor-killing functions, inflammatory cytokines production such as TNFα, IL-1β, IL-6, and IL-8, nitric oxide (NO) and reactive oxygen species (ROS), as well as improved priming capacities towards T cells *via* upregulated MHC class I and II presentation and associated co-stimulatory molecules (17). On the other extremity of this spectrum, “M2-like” alternatively activated macrophages present tumor-facilitating characteristics, characterized by secretion of immunosuppressive effectors such as TGFβ and IL-10, promotion of tissue remodeling and expression of inhibitory checkpoint molecules such as PD-1 (18). However, the phenotypic spectrum of TAM is much more complex than initially described. In breast cancer, TAM can express in the same cells a combination of M1-like and M2-like signature genes that correlate along the same activation trajectory (19), and TAM subsets that exert pro-angiogenic capacities *via* the expression of pro-angiogenic factors and vascular promotion (20), or favor the formation of pre-metastatic niches in breast cancer (21), have been identified.

A close relationship between TAM and the tumor-specific ECM network has been known for almost 40 years (22), with more recent studies reporting an increasingly complex crosstalk between these two components of the TME, comprising multiple layers of molecular and cellular cues reciprocally influencing both TAM biology and ECM composition. In this review, we will describe how the tumor-specific ECM can modify TAM phenotype, function and migration in breast cancer and how in turn TAM can influence the ECM network to favor tumor growth and spread. We will explore how the understanding of these mechanisms can be exploited to offer novel therapeutic solutions for cancers in need of novel treatments, drug resistant or poorly immune infiltrated “cold” tumors.

### ECM Favoring Macrophage Infiltration in the Tumor

The prognostic impact of the immune infiltrate in tumors has historically been defined by cell density (23). The density of the TAM infiltrate varies between different cancer types, but these cells are particularly abundant in breast cancers, where they can represent up to 50% of the tumor mass (24). Correlative data suggest an association between the composition of the tumor-specific ECM and TAM infiltrate. For example, a higher deposition of hyaluronan, a glycosaminoglycan of the ECM, correlated with higher macrophage counts and poor outcome in a cohort of 278 people with breast cancer, regardless of their tumor subtype (25, 26). Moreover, ECM stiffness and activation of TGFβ signaling, classically associated with fibrosis, both positively correlated with the number of macrophages at the invasive front in 20 breast cancer patients (9). Similar associations have also been observed in murine models of breast tumorigenesis. For example, when Pten, a gene involved in tumor growth regulation, is inactivated in the stromal fibroblasts of mice mammary glands, in MMTV-ErbB2/neu mice, the spontaneous tumorigenesis observed in mice expressing wild type levels of stromal Pten was decreased compared to mice lacking stromal Pten, a phenomenon associated with both collagen I deposition and increased macrophage infiltration (27). Furthermore, in a MMTV-PyMT/colla1*^tm1jae^* model of spontaneous mammary tumorigenesis, increased collagen deposition within the tumor was associated with higher TAM numbers, an effect dependent on COX2 expression, and in which COX2 blockade limited TAM and collagen levels (28). Similarly, constitutive expression of CCL2 in the mammary epithelium, which leads to increased macrophage infiltration, was associated with increased stromal deposition of collagen, that could elevate the risk of cancer development (29). Also, the overexpression of CCL2 by breast stromal cells transplanted into mouse mammary glands leads to enhanced TAM infiltration, concomitant with increased collagen expression. Both of these effects were ablated by depletion of CD11b expressing cells (30). These data suggest that tumors can manipulate the CCL2/CCR2 pathway to facilitate the infiltration of tumor prone collagen-producing macrophages.

**Figure 1:**
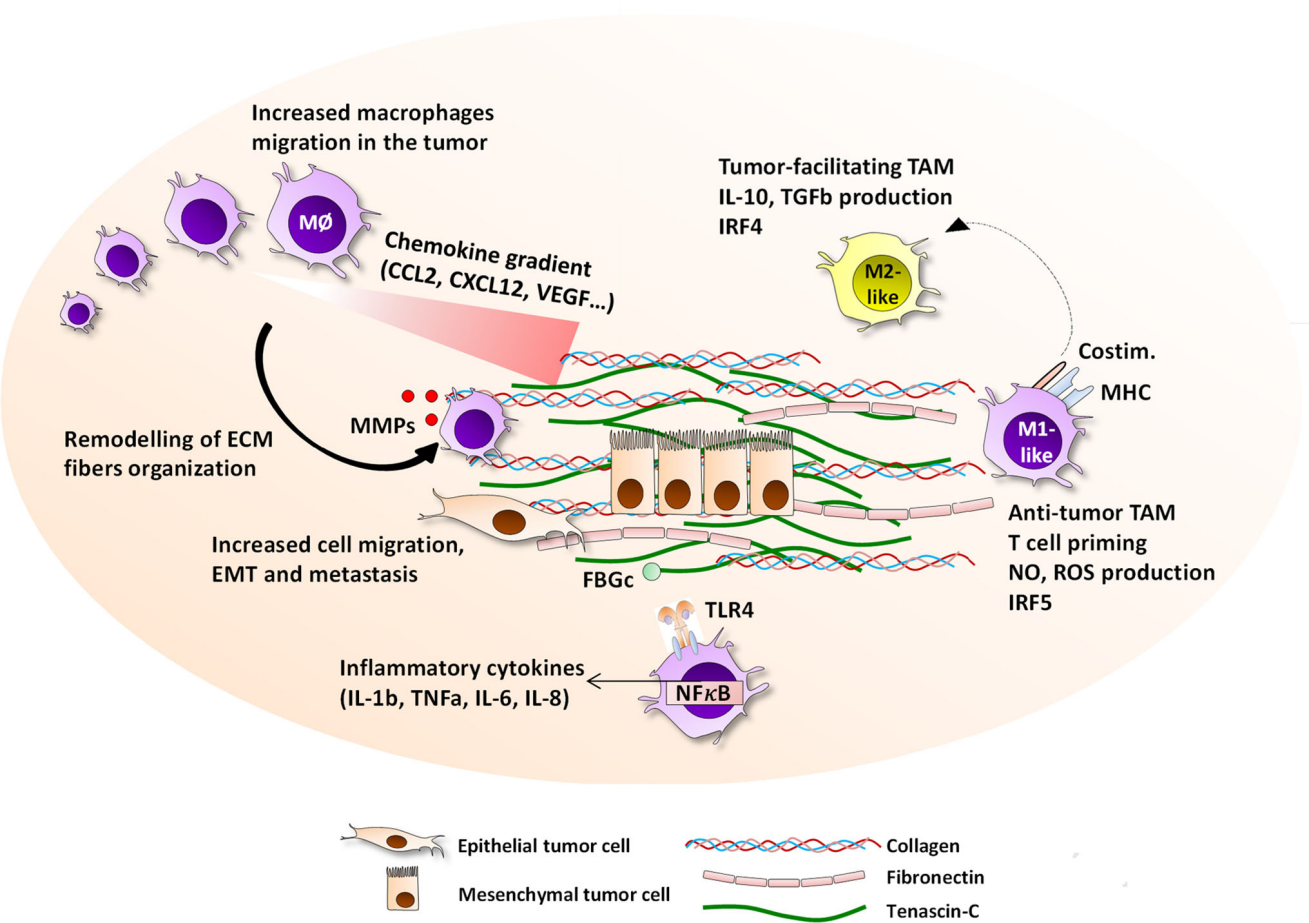
Interactions between the ECM and macrophages in the tumor microenvironment. The tumor-specific ECM network has a panel of possible interaction pathways with macrophages, all of which ultimately impact the evolution of cancer growth and prognosis. ECM molecules including tenascin-C, collagen, fibronectin, osteopontin, hyaluronan, versican, and thrombospondin, are highly upregulated in primary and metastatic breast cancer and embed epithelial tumor cells, and are produced by tumor cells, CAF, or immune cells. The ECM presence is associated with an increased migration of macrophages to the tumor site, with which they directly interact *via* the expression of integrins including αMβ2, α2β2, α2β1, αvβ5, or α9β1, or are guided by patterns of chemokine-matrix gradients. On site, macrophages are able to degrade the ECM fibers by secreting MMP2, 9, 13, and 14, and reorganize the collagen fibers. Together with their capacity to help cancer cell migration, intra- and extravasation, and initiating the EMT process, ECM help TAM contribute to accelerated metastasis. The ECM network is able to drive TAM towards either pro-angiogenic, anti-tumor M1-like or anti-tumor M2-like phenotype depending of the local contexture. Moreover, the EDA and FBG domains of fibronectin and tenascin-C respectively are TLR4 ligands that can trigger inflammatory responses in myeloid cells.

Together these studies demonstrate a positive correlation between TAM density, ECM remodeling and tumor progression, although it is difficult to distinguish cause and effect. The underlying reasons for altered TAM density are also not known; it is possible that this phenomenon may result directly from higher TAM infiltration, but could also arise from changes in monocyte infiltration and subsequent differentiation, and/or changes in monocyte and macrophage survival (**Figure 1**).

**Figure 2:**
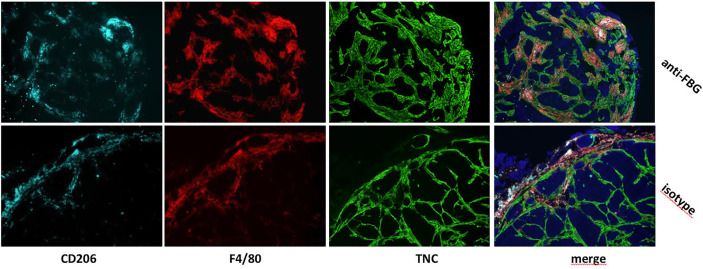
*In vivo* blockade of the FBG domain of tenascin-C diminishes TAM numbers and restrict their presence to tumor edge in a mammary tumor model. Mammary tumors from mice that were treated with a blocking anti-FBG antibody (upper panels) or a control isotype (lower panels) were collected 21 days after engraftment and stained with anti-CD206 (light blue), anti-F4/80 (red), and anti-TNC (green) antibodies.

More information has come from colocalization studies, which imply direct matrix-TAM interactions within the TME in experimental breast cancer. For example, using an orthotopic mammary tumor model, in which grafted tumor cells were engineered to express high or low levels of tenascin-C, we observed not only more numerous TAM in tenascin-C high tumors, but that TAM were exclusively present inside “tracks” formed by tenascin-C deposition. Treatment of mice with function blocking anti-tenascin-C antibodies caused TAM to accumulate at the edge of the tumor, compared to higher numbers within the tumor stroma in untreated mice (**Figure 2**) (31). These data indicate the capacity of ECM molecules to promote TAM infiltration during tumorigenesis, and demonstrate a role for the tumor specific-matrix in controlling the spatial positioning of TAM once within the TME. Conversely, matrix molecules may also restrict TAM infiltration; for example, blockade of the EDA domain of fibronectin in a mouse colon cancer model reduced tumor growth and led to increased infiltration of macrophages in the tumor (32), with a direct interaction of Fn-EDA with macrophages demonstrated by immunofluorescence (33). These data reveal not only the versatility of the effect of ECM molecules on TAM infiltration, but also indicate that this function may be limited to specific domains of these large multimodular molecules. However, little is known at the molecular level as to if, and if so how, these matrix molecules directly control TAM migration and positioning in the breast TME, or whether changes in the matrix indirectly affect immune cell infiltration.

Several cellular and molecular pathways have been brought forward from studies outside of breast cancer to explain how matrix molecules can interact with macrophages to promote their infiltration. TAM adhere directly to fibronectin and collagens in the ECM *via* integrins such as αMβ2 and α2β2 (34). These interactions have been shown to contribute to TAM motility and functions within breast (35), lung and colon (36), and prostate (37) cancers. However, binding to other matrix components, using other integrins, may also play a role. For example αvβ5, expression by TAM in glioblastoma serves as a receptor for the matrix glycoprotein osteopontin, whose interaction provides a chemotactic signal that is essential for macrophage recruitment (38). In addition to cell-matrix adhesion directly mediating TAM infiltration, the ECM also serves as a reservoir for soluble factors including chemokines. Chemokine-matrix interactions, in particular chemokine binding to matrix-resident glycosaminoglycans, are important in controlling not only local concentrations of these soluble factors, but also their oligomerization state, resistance to proteolysis, activity and signaling capabilities (39, 40). As such changes in the tumor-specific matrix may alter the capacity to bind and retain secreted molecules classically produced by tumor cells, thus impacting cell infiltration. For example, CCL2, a major player responsible for monocyte/macrophage infiltration into tumors, is known to rely on glycosaminoglycans within the extracellular matrix to effectively signal (41, 42). Moreover, other matrix-chemokine mediated mechanisms may also be at play in modifying TAM migration. In a mouse model of bladder cancer, versican, a large extracellular matrix proteoglycan, enhances lung metastasis in a manner dependent on the presence of CCL2 and of macrophages in the TME (43). One could argue that signals from the TME could induce parallel production of both versican and CCL2 by tumor cells, which signals could synergize in favor of metastasis colonization.

Together, these studies demonstrate that in breast cancer, there is a correlation between changes in expression levels of ECM molecules such as hyaluronan, collagen, fibronectin or tenascin-C with intratumoral TAM density, linked to disease outcome (**Table 1**). Moreover, data are emerging that the tumor-specific ECM creates sub-tumoral niches or tracks to control the distribution of TAM within the TME. Studying other cancer models has revealed a number of proposed mechanisms to explain how the ECM network may promote TAM infiltration, which includes providing an adhesive substrate for cells to attach to, and move along, and the patterning of TAM-attracting chemokines to provide migration permissive gradients. Their evaluation offers new clues for a better understanding of the mechanisms at stake in breast cancer.

**Table 1:** TAM and ECM interactions described in breast cancer studies.

Effect	Disease or model	ECM	Effect of ECM	Ref
**ECM favoring macrophage infiltration in the tumor**				
Correlation TAM infiltration and ECM	Human breast cancers	COLL	TAM number, TGFβ signaling and ECM stiffness all positively correlate	(9)
	MMTV-ccl2 model	COLL	CCL2 epithelial overexpression leads to increased TAM and increased COLL	(29)
	Human breast cancers	HA	HA and TAM correlate with a poor outcome	(25)
	MMTV-ErbB2/neu model	COLL	Inactivation of Pten leads to COLL remodeling and TAM infiltration	(27)
	MMTV-PyMT/colla1*^tm1jae^* model	COLL	Increased COLL deposition leads to higher TAM number in the TME	(28)
Colocalization of TAM and ECM	NT193 TNC^+/-^ cells orthotopic	TNC	TAM in the TME are trapped in TNC tracks	(31)
Linked TAM infiltration ECM	CCL2^+/-^ cells orthotopic	COLL	CCL2-attracted TAM directly lead to stromal COLL deposition in tumor	(30)
**ECM as a modulator of TAM phenotype in the TME**				
ECM driving M2 TAM	4T1 cells orthotopic	COLL	IL-6 and COLL drive TAM towards wound healing phenotype	(44)
	NT193 TNC^+/-^ cells orthotopic	TNC	TNC drives pro-tumoral M2-like TAM phenotype *via* TLR4-FBG interaction	(31)
ECM driving pro- angiogenic TAM	4T1 cells orthotopic	FN	TAM drive metastasis by recruiting VEGFR+ myeloid cells and promoting FN expression	(21)
	Breast carcinoma cells, breast xenograft	HA	High molecular weight HA drives pro-angiogenic behavior in breast TAM	(45)
**Macrophages as shapers of the tumor ECM**				
TAM reorganize collagen fibers	E0771 cells orthotopic	COLL	TAM reorganize COLL fibers to favor metastasis	(46)
TAM as ECM producers	Her2^+/-^ and ccl5^+/-^ cells orthotopic	COLL	CCL5 leads to the recruitment of TAM expressing COLL	(47)
	4T1 cells orthotopic	OPN	MDSC drive metastasis and immune suppression by producing OPN	(48)
**The regulation of EMT and metastasis by ECM and TAM**				
Cell migration promoting TAM	MMTV-PyMT model	COLL	TAM by COLL-rich tumor border support tumor cell intravasation	(49)
	4T1 cells orthotopic	SPARC	TAM-derived SPARC favors metastasis *via* integrin-dependent tumor cell invasion	(50)
EMT promoting TAM	MMTV-PyMT model	VSC	Myeloid cells-derived versican drives EMT and favor lung metastasis	(51)
	SPARC^+/-^ breast cancer cells	SPARC	Tumor cells derived SPARC induces EMT *via* the immunosuppressive functions of MDSC	(52)
**The need for CAF in the TAM-ECM relationship**				
CAF-TAM-ECM crosstalk	MMTV-PyMT model	COLL	Fibroblast-derived FAP signaling cleaves collagen and increases TAM adhesion	(53)
	NT193 TNC^+/-^ cells orthotopic	TNC	Tumor-derived TNC switches TAM phenotype, but not CAF-derived TNC	(31)

COLL, collagen; FN, fibronectin; HA, hyaluronan; OPN, osteopontin; SPARC, secreted protein acidic and rich in cystein; TNC, tenascin-C; VSC, versican.

### ECM as a Modulator of TAM Phenotype in the TME

As the complexities of macrophage biology continue to emerge, it is clear that moving beyond a simple consideration of intra-tumoral TAM density, to take into account the nature of the TAM infiltrate, is essential. If the tumor-specific ECM network is able to modulate the infiltration of myeloid cells in the tumor tissue, it can also directly impact their polarization and activation status, by driving them either towards tumor-facilitating or anti-tumor phenotype. *In vitro*, the impact of matrix on cultured macrophages and macrophage cell lines has been well documented, and most report that the ECM drives an M2-like phenotype. This was first noted in a historical paper from 1983, where Kaplan demonstrated the immunomodulatory role of collagen by cultivating human primary monocytes on collagen, which blunted their capacity to kill cancer cells (22). Similarly, overexpression of the anti-inflammatory transcription factor ATF3 (activating transcription factor 3) in RAW 264.7 macrophage cell line lead to an upregulation of tenascin-C, which was directly responsible for subsequent M2 differentiation and increased migration (54), whilst a hyaluronan and collagen mix drove the upregulation of the hyaluronan receptor CD44 in THP-1 macrophage cell line, together with the upregulation of a group of M2-related genes like CD163, IL-10 and CCL22 (55).

These studies support the idea that in breast cancer, tumors can manipulate ECM production to highjack the immune infiltrate, and switch the phenotype of TAM from efficient tumor killing cells to tumor-facilitating cells, further helping the tumor to thrive. But driving macrophages towards M2-like polarization is not the only impact induced by the ECM; these external signals can also affect another major pro-tumoral function of macrophages in tumors - to enhance or accelerate the neoangiogenesis required for the growth of cancer (56). The culture of macrophages with conditioned media of breast carcinoma cells in the presence of high molecular weight hyaluronan lead to an increased production of angiogenic factors such as VEGF, and increased endothelial cell migration. In line with this, macrophages primed with high molecular weight hyaluronan increased the number of blood vessels in breast carcinoma xenograft models (26, 45). Similarly, the chemical inhibition of CYP4A (Cytochrome P450 4A) in TAM in mouse models of breast cancer skewed their phenotype away from M2-like, and decreased the recruitment of VEGFR1+ myeloid cells and the expression of fibronectin by fibroblasts, altogether contributing to the metastatic process (21).

The molecular mechanisms by which the tumor-specific ECM may orientate the activation and polarization of macrophages and the immune response in the TME includes a variety of secreted factors, in particular those controlled by toll-like receptors (TLR) and NF-κB dependent inflammatory signaling. Indeed, it has been known for a long time that not only infectious triggers can drive a TLR-dependent responses in myeloid cells expressing these receptors, but endogenous triggers, or DAMPs (damage associated molecular pattern), also activate these cells during “sterile” inflammation. DAMP-mediated TLR signaling is involved in the pathogenesis of inflammatory diseases such as auto-immune diseases, atherosclerosis and cancer, where it can initiate and maintain a deleterious chronic immune response (57). ECM domains that are highly expressed in cancer have been identified as TLR-engaging DAMPs, and are likely to be involved in the skewing of the TAM phenotype and functions. For example, the fibrinogen-like globe (FBG) domain of tenascin-C was identified more than 10 years ago as a ligand of TLR4, and is able to engage aberrant inflammatory responses in TLR4-expressing myeloid cells *via* the release of TNFα, IL-6, and IL-8 (58), that are distinct from LPS-dependent responses (59), prolonging inflammation in arthritis models. Recently, we used an orthotopic grafting murine model of breast cancer to demonstrate that the tumor-derived tenascin-C is able to switch the phenotype of TAM towards a M2-like, pro-tumoral polarization, in a FBG/TLR4 dependent fashion (31). The triggering of this pathway generated a deleterious inflammatory contexture that helped the tumor escape from immune surveillance and supported a pro-metastatic environment, demonstrating a role for the TLR4 engagement by tenascin-C for tumor growth and spread. Similarly, a pro-tumorigenic role for versican-mediated TLR activation has been reported. When produced by lung lewis carcinoma tumor cells, versican acts as a ligand for TLR2 and TLR6 expressed by macrophages, generating a strong TNFα response from these cells, and ultimately acting as a help for metastasis (60), *via* mechanisms that could include TNFα-dependent stimulation of cancer cells proliferation, intravasation and extravasation.

However, little is still known about the impact of ECM-driven inflammation on macrophages phenotypes and functions in breast cancer, including the role of other TLR-binding ECM proteins and the importance of the local microenvironment. It is however noteworthy that matrix-mediated TLR ligation is not always tumor supportive. For example, fibronectin, in which the alternatively spliced EDA domain is also able to bind TLR4 (61), activates inflammatory responses in TLR4-expressing cells. This has led to the use of the EDA domain as an adjuvant for a protein vaccine derived from HPV (human papillomavirus) against HPV+ cervical carcinoma, which was able to generate antigen-specific CD8 T cells and eradicate tumors (62) *via* the activation and maturation of myeloid cells, hence triggering anti-tumor responses. Another example of matrix being able to modulate the orientation of tumor immunity one way or another is thrombospondin-1. Thrombospondin-1 can indeed generate enhanced expression of TNFα in bone marrow derived macrophages upon triggering of its receptor CD36, *via* signaling mediated by TLR4 and NF-κB (63), and the addition of exogenous thrombospondin-1 to a murine macrophage cell line *in vitro* blocked IL-10 production induced upon ionizing radiations (64), suggesting a tumor-facilitating role. But on the other hand, thrombospondin-1 can also exert a protective activity against carcinogenesis *in vivo*, as its absence during skin carcinogenesis limits cancer growth *via* its anti-inflammatory properties, including decreasing the levels of IL-6 and IL-12 and limiting the local infiltration of neutrophils and macrophages (65), highlighting a dual and probably context-dependent role of this protein during carcinogenesis.

Matrix-mediated pro- and anti-tumoral effects may be accounted for by the fact that different stimuli have different effects, despite using the same receptor, or receptor family. However, for one TLR4 ligand, the story is more complex, exerting distinct effects depending on the disease model or even the cellular source. Whilst we showed that tumor-derived tenascin-C drives M2 macrophage polarization in experimental mammary tumors, contradictory findings to ours have been found in glioblastoma. The absence of expression of CD47 on tumor cells increased the expression of tenascin-C in the TME, which in turn triggered TLR4-dependant inflammation in macrophages, characterized by high levels of TNFα secretion and activation of STAT-3 dependent signaling, together with an increased phagocytosis of tumor cells, suggesting an anti-tumor role for this ECM molecule in this model (66). Data from mouse models of cardiac pathologies show that up-regulation of tenascin-C during disease was associated with the shifting of macrophage phenotype towards M1-like *via* the engagement of TLR4 (67). However in hepatocellular carcinoma, Nong et al. demonstrated that macrophage-derived TNFα induces the production of tenascin-C by cancer cells in an NF-kB dependent pathway, promoting cell migration and tumor aggressiveness (68). Indeed, when we deleted host tenascin-C we found that, in contrast to deletion of tumor-derived tenascin-C, this resulted in diminished M1-like macrophage behavior (31). These data suggest that whilst host-derived matrix can be used to trigger TLR-mediated anti-tumoral immune responses, tumor-derived matrix can trigger TLR-mediated tumor supportive phenotypes, which may explain why pre-clinical global TLR4 blockade has provided mixed results to date.

These data highlight how the cell source of the matrix, the local microenvironment and specific tissue pathology can influence the pro- or anti-tumoral role of the ECM on immunity. Moreover, it is however important to note that the *in vitro* impact of any single matrix molecule on macrophage phenotype may be more complex than a black and white dichotomy, as highlighted by Huleihel et al. This group exposed macrophages to ECM bioscaffolds (69) and observed that macrophages turned either M1-like or M2-like depending on the tissular origin of the ECM. Moreover, pre-activation of macrophages with IFNγ and LPS lead to a decrease of inflammatory responses in all ECM stimuli tested, altogether indicating that not only the ECM network composition can influence macrophage activation status, but that the inflammatory contexture may also orientate their polarization.

Together, these studies reveal the role of the immunomodulatory properties of the ECM to be a double edged sword in the shaping of the immune response, as ECM molecules can drive an M2 phenotype in TAM as well as triggering TLR and NF-κB dependent inflammatory responses based on the specific cues of the local cellular and tissular microenvironment. Understanding the mechanisms by which the ECM network shape the TAM phenotype may offer clues about events occurring during breast cancer. Although it is likely that the levels of ECM expression and the number of macrophages may modulate these responses, the cellular source of ECM molecules, which alternatively spliced domains can be differentially expressed by different cell types, is also an important factor to take in account for the capacity of ECM to modulate macrophages, and will be discussed below.

### Macrophages as Shapers of the Tumor ECM

Whilst the tumor-associated matrix is able to modify macrophage infiltration, organization and phenotype, in turn, macrophages are also capable of directly modulating the organization and composition of the ECM network. This phenomenon has been well described in particular *via* the capacity of macrophages to secrete matrix metalloproteinases (MMP), enzymes that are able to degrade ECM proteins and that are key determinants for facilitating cell migration (70). In breast cancer, MMP-2, 9, 13 and 14 are involved in a broad spectrum of actions including the remodeling of the ECM, cell migration and metastasis, as well as neo-angiogenesis (71). Several studies further suggest that macrophages have a crucial role in the organization of the ECM. In CSF-KO mice, where numbers of circulating and mammary glands resident macrophages are greatly reduced due to their dependency on CSF, mammary tissue levels of collagen were unaffected, however collagen fibrillogenesis into long fibers was impaired (72). Moreover, the depletion of TAM dramatically altered collagen fibrillar microstructure in the tumor, changes that were associated with an increase in the number of lung metastases in an orthotopic engraftment breast tumor model (46). This could be explained by the involvement of macrophage-derived MMPs, contributing to the degradation of the matrix and as a consequence help for cancer cell intravasation and extravasation. These data suggest a role for TAM in the organization, or re-organization, the matrix, but other studies show that TAM may also play a role in *de novo* synthesis of the tumor specific ECM.

Macrophages can directly secrete ECM components. For example, this was demonstrated by an RNA expression study in primary human cells, showing that most myeloid cells are able to secrete tenascin-C upon activation (73). Similarly, macrophages are also able to produce different types of collagen upon a TLR4-dependent activation (59), further demonstrating the importance of TLR triggers is this crosstalk. Moreover, the presence of IL-6 and collagen in a triple negative mammary tumor model drove TAM towards a “wound healing” phenotype characterized by the production of effectors of the inflammatory phase of wound healing IL-1β, IL-6 and osteopontin in that in this context facilitated the trans-endothelial migration of tumor cells (44). Whilst a link between macrophage residence in the TME and matrix synthesis in the breast remains to be further explored, data are available from other tumor sites. For example, using an orthotopic model of colorectal carcinoma, Afik et al. showed that the ECM composition in tumors is markedly different in TAM high or deficient tumors as TAM activate matrix remodeling programs upon their differentiation from monocytes (74). In particular, TAM were able to express unique ECM and ECM-associated genes in the TME, including collagen type I VI and XIV, collagen synthesis and assembly as well as matrix cross linkers gene sets, leading to an impaired deposition, cross-linking and linearization of the matrix in presence of TAM, and altogether shaping the tumor invasiveness (74). The idea has been broached that TAM have a unique impact on ECM remodeling compared to other macrophage subsets. This was investigated in a study showing that TAM from ovarian carcinoma are remarkably similar at the transcriptomic and protein expression levels to resident peritoneal macrophages, sharing features such as phagocytic and antigen presentation capabilities levels (75). However, TAM had a non-overlapping gene expression signature not shared with monocyte-derived macrophages and resident peritoneal macrophages, mainly composed of matrix-remodeling genes and collagen fiber organization. These data indicate that these macrophage subpopulations acquire particular capacities to manipulate the ECM in tumors (75). How these cells acquire these novel capabilities remains unclear but this re-programming may be induced by tumors cells in order to aid tumor escape from immunosurveillance.

In a model of mammary tumor engraftment by tumor cells with a conditional Her2 downmodulation inducible by doxycycline which leads to tumor regression, the following tumoral recurrence is associated with an increased tumor production of CCL5, a chemokine involved in many aspects of tumor progression in breast cancer (76). The authors link this secretion with the recruitment of CCR5 expressing macrophages that express genes of collagen and collagen-deposition factors such as procollagen C-endopeptidase enhancer 1 and asporin (47), suggesting that the CCL5/CCR5 pathway can be manipulated by tumors to provoke macrophage to directly deposit collagen into the tumors, favoring their recurrence.

The functional implications of TAM-derived ECM networks are also emerging, with data showing that immune cell-mediated matrix synthesis directly contribute to tumor growth. For example, in mouse models of mammary tumors cell lines orthotopic engraftment, the use of osteopontin wt or ko mice showed that myeloid derived suppressor cells (MDSC) exerted a more immunosuppressive impact at the metastatic site in presence than in absence of osteopontin (48). In non-small cell lung cancer, the concomitant tissue detection and quantification of macrophages and osteopontin revealed that osteopontin produced by TAM is also associated with progression and poor survival (77) and in colorectal cancer co-culture models, the expression by cancer cells of osteopontin receptor CD44 drives its production by TAM, which in turn favors tumor cells tumorogenicity (78). On the contrary, Szulzewsky et al. showed that glioblastoma-associated monocytes are the main producers of osteopontin in the TME and that it exerts an anti-tumor effect, as opposed to tumor-derived osteopontin which has little impact (79). This suggests that TAM-derived osteopontin can be used as a communication tool by macrophages to interact with cancer cells and impact the TME. This idea of an intermediate signal used by macrophages to shape the ECM in breast cancer can also be applied to TGFβ, which not only can be produced by tumor-facilitating M2-like TAM and is a key regulator of carcinogenesis in tumors such as breast cancer (80), but is also a key signal involved in fibrosis and ECM production (81). Moreover, TGFβ in conjunction with tenascin-C are associated with an epithelial to mesenchymal transition (EMT) of breast cancer cells (82), and stimulation of macrophages by TGFβ force them to produce type VI collagen (83). Finally, although not in models of tumorigenesis, but during experimental dermal remodeling, CCR2 expressing macrophages directly degraded collagen and fibrins, and the addition of GM-CSF selectively enhanced their collagen endocytosis capacities, likely *via* the proliferation-inducing capacities of this cytokine (84). These data suggest an involvement of the CCL2/CCR2 axis in matrix remodeling and tissue repair mechanisms, in favor of a positive reinforcement loop between the ECM and macrophages; it is for example known that collagen degradation products can play a chemotactic role towards macrophages, which may contribute to their recruitment to the tumor site (85).

These data demonstrate how macrophages can modify the composition and the organization of the ECM network in tumors by producing specific ECM or ECM associated components or by reshaping collagen fibers, showing that TAM-ECM crosstalk occurs in both directions.

### The Regulation of EMT and Metastasis by ECM and TAM

One of the best studied roles of the ECM network in breast cancer is its contribution to driving metastatic transition (86). The role of TAM in this process have also been extensively studied *via* their capacity to secrete matrix metalloproteinase MMPs that are critical for the digestion of ECM prelaminar to the migration of cancer cells outside the primary tumor site (87), and this has been reviewed elsewhere (88, 89). However, other mechanisms of interplay between TAM and ECM have also been reported in breast cancer models, such as in driving epithelial to mesenchymal transition (EMT). For example, versican, a matrix proteoglycan, is produced by monocytes in pre-metastatic lesions where it aids breast cancer cell transformation in MMTV-PyMT mice (51), whilst expression of the ECM glycoprotein SPARC (secreted protein acidic and rich in cysteine) by breast tumor cells induces EMT dependently on the presence of MDSC (52) in breast cancer cell engraftment, and expression of SPARC by macrophages induces cell migration and metastasis in triple-negative breast cancer cells engraftment (50). Moreover, Wyckoff et al. elegantly used intravital microscopy in a MMTV-PyMT model to show that the cancer cell intravasation observed by live imaging in mammary tumors was helped by perivascular macrophages that accumulated by collagen-rich tumor border in association with the EGF and CSF-1 pathways (49). Together, these data provide clues for a better understanding of one of the tumor-facilitating roles of the ECM-TAM collaboration, which is the promotion of early events crucial for metastasis in epithelial cancers including EMT and cancer cell migration.

### The Need for CAF in the TAM-ECM Relationship

The presence of a specific ECM network within tumors is the result of the contribution of many cellular players including not only tumor cells and immune cells, but also CAF. CAF are critical for breast cancer evolution and important producers of extracellular matrix in tumors (90, 91). The interactions between CAF and TAM are becoming increasingly well characterized, for example co-culture of monocytes with pancreatic tumor cells with CAF drives monocytic differentiation into cells with an M2-like macrophage phenotype (92). Moreover, CAF and TAM crosstalk can synergize to increase the invasiveness of the tumor by increasing cell mobility, as well as favoring neoangiogenesis programs (93). Indeed, a role for the ECM in this intercellular interplay is also emerging; endogenous fibroblasts and TAM may also matrix molecules as a means to communicate with one another in the breast TME. For example, fibroblast synthesis of FAP (fibroblast activation protein), a membrane-bound serine protease that can cleave collagen fibers and thus increase TAM adhesion in an MMTV-PyMT breast cancer model (53). In our work, using a syngeneic orthotopic grafting model of breast cancer, the absence of tenascin-C production by tumor cells was compensated by CAF-derived tenascin-C, meaning that total tumor levels of this matrix protein were not altered when grafting tumors with high or low tenascin-C expression levels. However, tenascin-C derived from each cellular source had a dramatically different impact on the TAM phenotype; only tumor-derived tenascin-C could induce a pro-tumor TAM phenotype, as opposed to CAF-derived tenascin-C (31). These data together pinpoint CAF as contributors of the TAM-ECM crosstalk in the breast TME and potential target to block these pro-tumor processes.

## Conclusions and Perspectives

A growing number of studies report ever increasingly complex interactions between the ECM and the tumor myeloid compartment. These extend beyond the degradation of ECM by TAM-derived MMPs and integrin-mediated TAM-matrix cell adhesion. As discussed here, tumor-specific ECM and TAM crosstalk has multiple faces; the ECM can shape macrophage infiltration, positioning and phenotype, driving cells towards anti- to pro-tumoral, inflammatory, pro-angiogenic or pro-metastatic depending on the local tissue contexture, whilst macrophages can systematically modify the organization of ECM fibers, as well as the composition of the ECM network (**Figure 1**, **Table 1**). The coordination of the ECM–TAM crosstalk ultimately impacts the orientation and strength of the innate and adaptive anti-tumor immune response, and thus modulates the rate of the primary tumor growth and metastasis. The importance of these interactions for cancer evolution therefore represents a pool of promising biomarkers and myriad of potential therapeutic targets for new anti-cancer immunotherapies in breast cancer.

Moreover, the interaction between the ECM and TAMs is not static, but constantly changing. As the tumor develops, grows, and spreads, changes in the tumor microenvironment brought by cooperative anabolism and catabolism of CAFs, proliferating tumor cells and infiltrating immune cells contribute to dynamically modify the biochemical and mechanical signals mediated by the ECM that shapes cell behavior. At present, we have a limited understanding of the complexities of this evolving TAM-ECM relationship, despite the recent increase in the number of interactions being discovered and functionally characterized, and with it the perspective of novel biomarkers (94). Furthermore, more detailed investigation into pathways that are universal amongst tumors, compared to tumor-, or tissue-, specific changes will be of importance. This approach may indeed shape our understanding of how the crosstalk between ECM and TAM may have a different impact on cancer evolution depending on disease stage, tumor type and contexture as well as articulation with co-treatments, and become critical for the success of new immunotherapies.

The field of immunotherapies in general, and in cancer in particular, is indeed evolving in giant strides, as represented by the success of T-cell targeting immune checkpoints inhibitors anti-PD1, anti-PD-L1 and anti-CTLA4, that revolutionized treatment of cancer patients (95). But immune checkpoints inhibitors face limitations, due to induced resistance, lack of target expression, or lack of immune infiltrate in “cold” tumors (96, 97). In breast cancer, in particular, we see highly heterogeneous pathology (98), with a complex organization of the therapeutic landscape. Triple negative breast cancer (TNBC) that are defined by their lack of expression of progesterone receptor, estrogen receptors and Her2-neu particularly suffer from an absence of viable long-term therapeutic options, and although anti-PD-L1 immune checkpoints inhibitors have recently given promising results after a phase III clinical trial (99). To this end, and because of the importance of macrophages in this cancer, several macrophage-targeting options have been extensively explored, mainly including blocking the CCL2/CCR2 or the CSF1/CSFR1 pathways (100, 101), but they have faced mitigated success. Targeting specific domains of the tumor-specific ECM in order to reorient the immune response is in this context an emerging option which efficacy has been explored in different situations. In our model of mammary tumor expressing or not tenascin-C, the blockade of the TLR4-binding domain of tenascin-C, FBG, with a neutralizing antibody (102) lead to a reversion of the TAM phenotype towards a tumor-killing type, and was associated with decreased tumor growth and smaller lung metastases (31). Moreover, the therapeutic combination of this FBG-targeting therapy with an anti-PD-L1 antibody further diminished the tumor growth. Similar options were investigated against the EDA alternatively spliced domain of fibronectin, by developing a fusion protein with an anti-EDA sequence and calreticulin, an alarmin serving as a pro-phagocytosis signal towards myeloid cells. In a colon cancer model, its therapeutic use helped slow down tumor growth and this treatment worked synergistically with an anti-PDL1 antibody (32), confirming the interest for combining this type of matrix-targeting therapy modulating the macrophage innate response with T-cell targeting with immune checkpoints inhibitors to unlock different levels of tumor-associated immune suppression. Arribillaga et al. used the capacity of the EDA domain of fibronectin to activate myeloid cells *via* binding TLR4 (61) to develop an immunogenic antigen delivery tool by coupling EDA with proteins, activating dendritic cells, which were then able to effectively prime specific T cells (103), highlighting again the dual role of ECM domains of the activation of innate immune cells. Other options have been explored to use the immunomodulatory properties of fibronectin, including against the alternatively spliced EDB domain, which neutralizing sc-Fv fragment L19 was coupled with IL-2 to trigger immunogenicity (104). Thrombospondin-1 could also be used as a target, and its neutralization blocked the osteoclast differentiation from monocytes formation occurring during myeloma bone (105). Another way by which therapies investigate the blocking or enhancement of the ECM-macrophages interaction consists in targeting membrane-bound molecules serving as receptors for ECM or ECM-associated proteins. It is the case for CD47, a ligand of thrombospondin-1, which can be expressed by cancer cells and act as a “don’t eat me” signal towards macrophages, hence a good target for cancer (106). Anti-CD47 neutralization has shown promising anti-tumor properties by promoting the phagocytosis of cancer cells by TAM, and evidenced potential to synergize with other tumor-targeting therapies (107). Finally, the blockade of the interaction between ECM and TAM can be performed *via* integrin blockade. α4β1 in particular, is an integrin expressed by monocytes and necessary to their homing into the tumor as well as a receptor for the EDA domain of fibronectin (108) and its blockade on TAM limited the macrophage-induced neoangiogenesis (36), helping control the tumor growth. Finally, it is noteworthy that CSFR1 inhibitors were able to block the migration of macrophages induced by fibronectin (109). The exploration of how macrophages and the matrix work together to shape the TME provides a large panel of pathways that leads the tumor growth and spread, and the understanding of these signaling is still at its beginning. The majority of these studies report the tumor-facilitating roles of the TAM and ECM crosstalk, and these pathways constitute powerful and relatable predictive biomarkers for cancer evolution and metastasis. But they also provide invaluable indications for new pathways to target in novel therapy for cancers that are lacking options, like the TNBC. Preclinical studies suggest that targeting immunomodulatory domains of the ECM can be an efficient and safe way to reinstate an efficient innate immune response, and is working well as combination therapies, at a time where multi-hit therapy paves its way in the cancer therapeutic landscape.

## Author Contributions

CD and KM wrote the review. All authors contributed to the article and approved the submitted version.

## Conflict of Interest

KM is founder of, and consultant to, Nascient Ltd.

The remaining author declares that the research was conducted in the absence of any commercial or financial relationships that could be construed as a potential conflict of interest.

## Abbreviations

CAF, cancer-associated fibroblasts; CCL, chemokine ligand; CCR, chemokine receptor; DAMP, damage associated molecular pattern; ECM, extracellular matrix; EDA, Extra Domain A; FBG, fibrinogen-like globe domain; GM-CSF, granulocyte-monocyte colony stimulating factor; IFNγ, interferon gamma; IL, interleukin; M-CSF, macrophage colony stimulating factor; MMP, matrix metalloproteinase; NF-KB, nuclear factor kappa b; TAM, tumor-associated macrophages; TGFβ, transforming growth factor beta; TLR, toll like receptor; TNBC, triple negative breast cancer; TNFα, tumor necrosis factor alpha; TME, tumor microenvironment.
